# Five-year outcomes of next-generation sequencing implementation at a Brazilian public health system reference centre for rare diseases

**DOI:** 10.3389/fgene.2026.1850909

**Published:** 2026-07-29

**Authors:** Natana Chaves Rabelo, Maria Eduarda Gomes, Isabelle Correa de Moraes, Bianca Barbosa Abdala, Daltro C. Junior, Anneliese Barth, Patrícia Correia, Daniela Koeller Rodrigues Vieira, Fernanda Rolemberg, Naiara Gomes, Tatiana S. P. C. Magalhães, Dafne Dain Gandelman Horovitz, Juan C. Llerena, Sayonara Gonzalez

**Affiliations:** 1 Laboratório de Biologia Molecular/Medicina Genômica, Centro de Genética Médica José Carlos Cabral de Almeida & Centro de Referência para Doenças Raras – Instituto Nacional de Saúde da Mulher, da Criança e do Adolescente Fernandes Figueira (IFF) - FIOCRUZ, Rio de Janeiro, Brazil; 2 Unidade de Genética Clínica, Centro de Genética Médica José Carlos Cabral de Almeida & Serviço de Referência para Doenças Raras – Instituto Nacional de Saúde da Mulher, da Criança e do Adolescente Fernandes Figueira (IFF) - FIOCRUZ, Rio de Janeiro, Brazil; 3 Laboratório de Biologia Molecular/Medicina Genômica, Centro de Genética Médica José Carlos Cabral de Almeida, Instituto Nacional de Doenças Raras - INRaras (INCT), Rio de Janeiro, Brazil; 4 Departamento de Genética Médica, Faculdade de Medicina de Petrópolis – UNIFASE, Rio de Janeiro, Brazil

**Keywords:** genomics, health-equity, NGS, public health, exome, rare diseases

## Abstract

**Introduction:**

Rare diseases affect approximately 6%–7% of the Brazilian population, representing a significant public health challenge due to diagnostic delays and inequitable access to genomic services. This study evaluates a 5-year implementation of next-generation sequencing (NGS) at a Reference Service for Rare Diseases (RDRS) within the Brazilian Unified Health System (SUS), examining diagnostic performance and barriers to equitable access to genomic medicine.

**Methods:**

A cohort of 385 patients with suspected genetic disorders underwent clinical or whole-exome sequencing (CES/WES) between 2019 and 2024. Exome analyses were guided by standardized Human Phenotype Ontology (HPO)–based phenotypic characterization, resulting in an overall diagnostic yield of 38.7% (149/385 cases).

**Results:**

A total of 249 variants were identified across 165 genes, including 75 variants not previously reported in medical literature or public databases. Actionable secondary findings were detected in 3.2% of cases, involving pathogenic variants in genes related to cardiac disease, cancer predisposition, and anesthesia risk, underscoring the preventive potential of genomic testing.

**Discussion:**

The implementation of NGS in the Brazilian public health system is feasible and highly impactful, yielding a 38.7% diagnostic rate that significantly reduces the diagnostic odyssey for rare disease patients. Success was heavily dependent on a multidisciplinary approach, standardized HPO-based phenotypic characterization, and close clinical-laboratory integration. The identification of 75 novel variants highlights the genetic complexity of Brazil’s admixed population and the challenges of interpreting variants of uncertain significance (VUS) in underrepresented groups. Furthermore, the detection of actionable secondary findings demonstrates the broader preventive potential of genomic testing, underscoring the need for expanded local databases, genomic literacy among clinicians, and robust ethical frameworks for patient management.

## Introduction

Rare diseases (RDs) comprise approximately 7,000 distinct clinical conditions that profoundly affect the lives of patients and their families, most of which are caused by genetic abnormalities (Haendel et al., 2020). Collectively, these conditions affect more than 354 million people worldwide, including nearly 13 million Brazilians, and represent a significant burden on both families and the healthcare system. As congenital abnormalities constitute the second leading cause of infant mortality in Brazil, timely diagnosis is critical, as it directly influences diagnostic efficiency, healthcare expenditures, clinical management, genetic counseling, and long-term patient outcomes (Félix et al., 2025). Despite notable advances in genomic technologies, diagnostic delays within the Brazilian public healthcare system remain substantial, averaging 5–7 years. These delays stem largely from prolonged intervals between symptom onset and referral to specialized care and from the limited availability of comprehensive genomic testing, such as whole exome and whole genome sequencing (WES/WGS), across public health services, where many centers still rely on low complexity assays or restricted gene panels (Stranneheim et al., 2021; Malmgren et al., 2025; de Oliveira et al., 2023; de Oliveira et al., 2024).

Globally, whole exome sequencing achieves diagnostic yields ranging from 25% to 50%, prompting several high-income countries to incorporate standardized next-generation sequencing (NGS) approaches into their national healthcare systems (Mighton et al., 2026; Han et al., 2022; Ewans et al., 2022; Malmgren et al., 2025). Large scale initiatives, including the 100,000 Genomes Project in the United Kingdom (Cipriani et al., 2025), the All of Us research program in the United States (Bick et al., 2024), the Solve-Rare Diseases Consortium (Solve-RD) in Europe (Laurie et al., 2025) and the Saudi Human Genome Program (Monies et al., 2017), have demonstrated the clinical and economic benefits of population wide genomic implementation. In contrast, Brazil’s Unified Health System (SUS), despite its universal mandate, faces distinct barriers to NGS integration, including geographic disparities in genomic testing capacity, chronic underfunding of genomic technologies, and a growing shortage of medical genetics specialists. Even so, the incorporation of NGS into SUS represents a major advancement for individuals with rare diseases, particularly in a public health system constrained by structural inequities. Following the establishment of the Brazilian Policy for Comprehensive Care for People with Rare Diseases (Portaria GM/MS N° 199, 2014) national efforts have increasingly promoted the expansion access to genomic diagnostics through the accreditation and strengthening of Reference Services for Rare Diseases (RDRS).

Among these centers, IFF/Fiocruz has become a national leader in public sector genomic diagnostics. In 2019, the institution implemented NGS to expand its diagnostic capacity. During the early phase of implementation, molecular investigation focused on patients with suspected RASopathies (Chaves Rabelo et al., 2022). Initial analyses were conducted using sequencing platforms available at Fundação Oswaldo Cruz (https://plataformas.fiocruz.br/), supported by institutional investment in laboratory supplies and the training of specialized human resources, as well as strong support from institutional leadership. The initiative was subsequently expanded to include patients with other rare genetic disorders. Limited resources necessitated a targeted strategy centered on clinical exome sequencing (CES), which allowed testing to be tailored to specific clinical phenotypes and optimized available infrastructure. As case complexity and phenotypic heterogeneity increased, whole exome sequencing (WES) was incorporated into routine diagnostic workflows in 2023 to enhance diagnostic depth and improve coverage for genetically diverse conditions.

This study summarizes a 5-year experience implementing NGS within a public clinical genetics center, demonstrating how the integration of genomic technologies can improve diagnostic outcomes for patients with previously undiagnosed rare diseases. The adoption of CES and WES at IFF/Fiocruz substantially increased diagnostic capacity, reduced prolonged diagnostic odysseys, and enabled more accurate and timely clinical management, reinforcing the importance of accessible genomic medicine within universal healthcare systems.

## Methods

The study cohort included 385 patients evaluated at the IFF/Fiocruz Reference Center for Rare Diseases between 2019 and 2024. Participants were recruited through two main pathways: (1) multidisciplinary specialty clinics, comprising Medical Genetics, Pediatric Neurology, Neurosurgery, Dermatology, Critical Care, Fetal Medicine, Pulmonology, Neonatology, and Ophthalmology, and (2) external referrals from regional healthcare units in Rio de Janeiro.

Ethical approval was granted by the institutional research ethics committee (approval number 5.079.483), and written informed consent was obtained from all participants or their legal guardians.

### Clinical data collection and phenotypic standardization

During the early phase of implementation, molecular investigation focused on patients with suspected RASopathies and progressively broadened its scope to encompass a wider range of genetic disorders. Test requests were highly heterogeneous, ranging from specific diagnostic hypotheses to limited and non-standardized clinical descriptions. To address this variability, an internal workflow was established incorporating standardized Human Phenotype Ontology (HPO) terms into the evaluation process. When referring clinicians did not provide HPO terms at the time of sample submission, a team of medical specialists retrospectively reviewed medical records and assigned appropriate terms to ensure consistent phenotypic characterization.

To further standardize clinical categorization, each case was independently assessed by three medical specialists, who reached consensus on the most appropriate nosological group based on the reported clinical suspicion. Patients were classified into one of the predefined categories: skeletal and connective tissue; dermatological; neurodevelopmental disorders; congenital anomalies/syndromic; neuromuscular; inborn errors of immunity; renal; hematological; cardio-vascular; inborn errors of metabolism; congenital anomalies of the respiratory system; ocular/auditory; or other conditions not encompassed by these groups.

### Samples and molecular analysis

Peripheral blood samples were collected in EDTA-containing vacutainers from all probands and, when necessary for segregation analysis, from available first-degree relatives. Genomic DNA extraction was performed using the ReliaPrep™ Blood gDNA Miniprep System (Promega), following the manufacturer’s instructions.

Two complementary NGS approaches were employed. CES was performed using the TruSight One Expanded panel, targeting approximately 6,700 genes on the Illumina NextSeq 500 platform. Subsequently, WES was conducted using the Illumina Exome 2.0 Capture kit on the NovaSeq 6000 or NovaSeq X Plus platforms to improve diagnostic resolution for clinically heterogeneous disorders.

Bioinformatic processing was performed using the VarStation platform (VarsOmics, versions 2.0 and 3.0), through an automated analysis pipeline. Sequence reads were aligned to the GRCh37 or GRCh38 human reference genomes using BWA-MEM, followed by duplicate marking with Picard MarkDuplicates and base quality score recalibration using GATK BaseRecalibrator and ApplyBQSR. Single nucleotide variants (SNVs) and small insertions/deletions (indels) were identified using the GATK HaplotypeCaller algorithm and subsequently annotated through the platform’s SNV/Indel annotation workflow, integrating information from multiple population, disease, and prediction databases, including ClinVar and gnomAD. As this was a longitudinal study conducted over a 5-year period, database releases, annotation resources, and platform versions were periodically updated according to the versions available within the clinical pipeline at the time of analysis.

Variant interpretation and prioritization were performed considering phenotype-driven analysis based on Human Phenotype Ontology (HPO) terms provided at the time of test request, inheritance pattern, gene–disease correlation, population frequency, predicted molecular consequence, and available clinical classifications. Variants were required to meet a minimum coverage depth of 20× and a quality score > Q30 to be considered reliable. Variants not meeting these thresholds, but considered clinically relevant, were orthogonally confirmed by Sanger sequencing.

Variant interpretation was performed in accordance with ACMG/AMP guidelines, incorporating recommendations from the ClinGen Sequence Variant Interpretation (SVI) working group. In accordance with these recommendations, variants identified in genes with uncertain or insufficient evidence of disease association (genes of uncertain significance, GUS) were not considered for clinical reporting. Only variants in genes with established gene–disease associations were included in reports, using OMIM, Genomics England PanelApp green-level genes, and, more recently, ClinGen gene–disease validity curations as primary reference resources.

The assessment of secondary findings was restricted to WES cases, and the analyzed gene lists followed the periodic updates of the ACMG recommendations throughout the study period.

Segregation analysis by Sanger sequencing was performed whenever feasible for both recessive and dominant conditions, provided that parental or familial samples were available. Sanger reactions were carried out on an ABI 3730 DNA Analyzer using the BigDye Terminator v3.1 Cycle Sequencing Kit, following the protocol described by Otto et al. (2008).

Based on the phenotypic and molecular findings, individuals were classified into diagnostic categories adapted to the present study. Cases harboring pathogenic or likely pathogenic variants compatible with the expected inheritance pattern and consistent with the clinical phenotype were classified as diagnostic finding. Individuals presenting inconclusive molecular findings, including variants of uncertain significance (VUS) associated with compatible phenotypes, were classified as inconclusive. Cases without clinically relevant variants identified through genetic testing and/or lacking phenotypic compatibility were classified as no findings.

## Results

**FIGURE 1:**
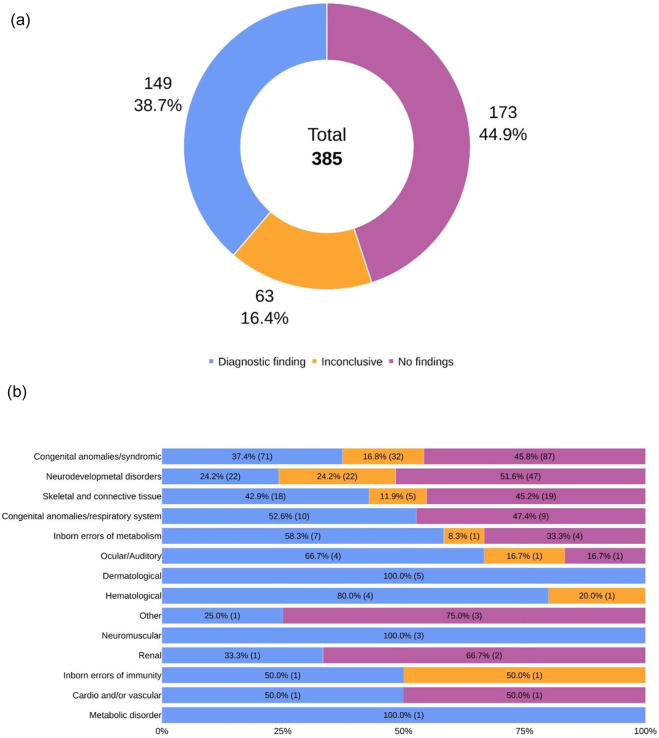
Exome sequencing diagnostic yield for rare diseases. **(a)** Overall diagnostic yield in the study cohort. **(b)** Diagnostic yield across nosological categories. Blue bars indicate diagnostic finding; orange bars represent inconclusive cases and purple bars no findings. Percentages were calculated relative to the total number of cases in each category (n).

The study cohort was predominantly pediatric, with 82% of patients aged ≤18 years old (316/386). Phenotypic characterization revealed a wide spectrum of clinical presentations, with congenital anomalies/syndromic disorders representing the most frequent category (49.4%), followed by neurodevelopmental disorders (23.6%) and skeletal/connective tissue disorders (10.9%) (Figure 1a).

For the three most representative nosological groups, the most frequently reported Human Phenotype Ontology (HPO) terms were examined (Table 1). Despite retrospective curation, standardized phenotypic annotation was unavailable for 33 cases, mostly referred from external services, which were therefore excluded from the HPO-based analysis. Among individuals with congenital anomalies/syndromic disorders, the most prevalent HPO terms were Intellectual disability (HP:0,001,249), Short stature (HP:0004322), and Developmental delay (HP:0,001,263). In neurodevelopmental disorders, the most common terms include Epilepsy (HP:0,001,250), Developmental delay (HP:0,001,263), and Intellectual disability (HP:0,001,249). For skeletal/connective tissue disorders, Joint hypermobility (HP:0,001,382) was the most frequently observed feature.

**TABLE 1:** Distribution of the ten most prevalent Human Phenotype Ontology (HPO) terms among individuals diagnosed with congenital anomalies/syndromic disorders, neurodevelopmental disorders and skeletal and connective tissue.

​	Congenital anomalies/Syndromic (n = 190)			Neurodevelopmental disorders (n = 91)			Skeletal and connective tissue (n = 42)		
Rank	HPO ID	Phenotype	Frequency	HPO ID	Phenotype	Frequency	HPO ID	Phenotype	Frequency
1	HP:0,001,249	Intellectual disability	22.1% (42)	HP:0,001,250	Epilepsy	30.7% (28)	HP:0,001,382	Joint hypermobility	11.9% (5)
2	HP:0004322	Short stature	18.4% (35)	HP:0,001,263	Developmental delay	25.5% (23)	HP:0000023	Inguinal hernia	9.5% (4)
3	HP:0,001,263	Developmental delay	14.7% (28)	HP:0,001,249	Intellectual disability	18.6% (17)	HP:0002650	Scoliosis	9.5% (4)
4	HP:0000664	Synophrys	13.6% (26)	HP:0200134	Epileptic encephalopaty	16.4% (15)	HP:0000218	High palate	9.5% (4)
5	HP:0000316	Hypertelorism	10.5% (20)	HP:0,001,252	Hypotonia	10.9% (10)	HP:0009760	Antecubital pterygium	9.5% (4)
6	HP:0,001,250	Epilepsy	10% (19)	HP:0000252	Autism	9.8% (9)	HP:0004322	Short stature	9.5% (4)
7	HP:0000527	Long eyelashes	10% (19)	HP:0000252	Microcephaly	9.8% (9)	HP:0000465	Webbed neck	7.5% (3)
8	HP:0000767	Pectus excavatum	7.8% (15)	HP:0002376	Developmental regression	8.7% (8)	HP:0000470	Short neck	7.5% (3)
9	HP:0000369	Low-set ears	7.8% (15)	HP:0002650	Scoliosis	7.6% (7)	HP:0,001,263	Developmental delay	7.5% (3)
10	HP:0000494	Downslanted palpebral fissures	7.3% (14)	HP:0000256	Macrocephaly	7.6% (7)	HP:0002652	Skeletal dysplasia	7.5% (3)

Among the 385 sequenced cases, 144 underwent CES and 241 underwent WES. Across these analyses, 249 variants were identified in 165 genes, including 75 variants not previously reported in medical literature or public databases. These novel variants were submitted to ClinVar and are available via the laboratory’s submissions page (https://www.ncbi.nlm.nih.gov/clinvar/?term=Laboratorio+de+Biologia+Molecular%2FMedicina+Genomica+-+IFF%2FFiocruz). Overall, 149 cases were classified as positive, defined by the presence of pathogenic or likely pathogenic variants consistent with the reported phenotype and inheritance pattern; while 174 yielded negative results and 62 were considered inconclusive due to the presence of at least one variant of uncertain significance (VUS) plausibly related to the clinical presentation (Figure 1b). These findings correspond to an overall diagnostic yield of 38.7%. By testing modality, CES achieved a diagnostic yield of 50%, whereas WES yielded 31%.

**FIGURE 2:**
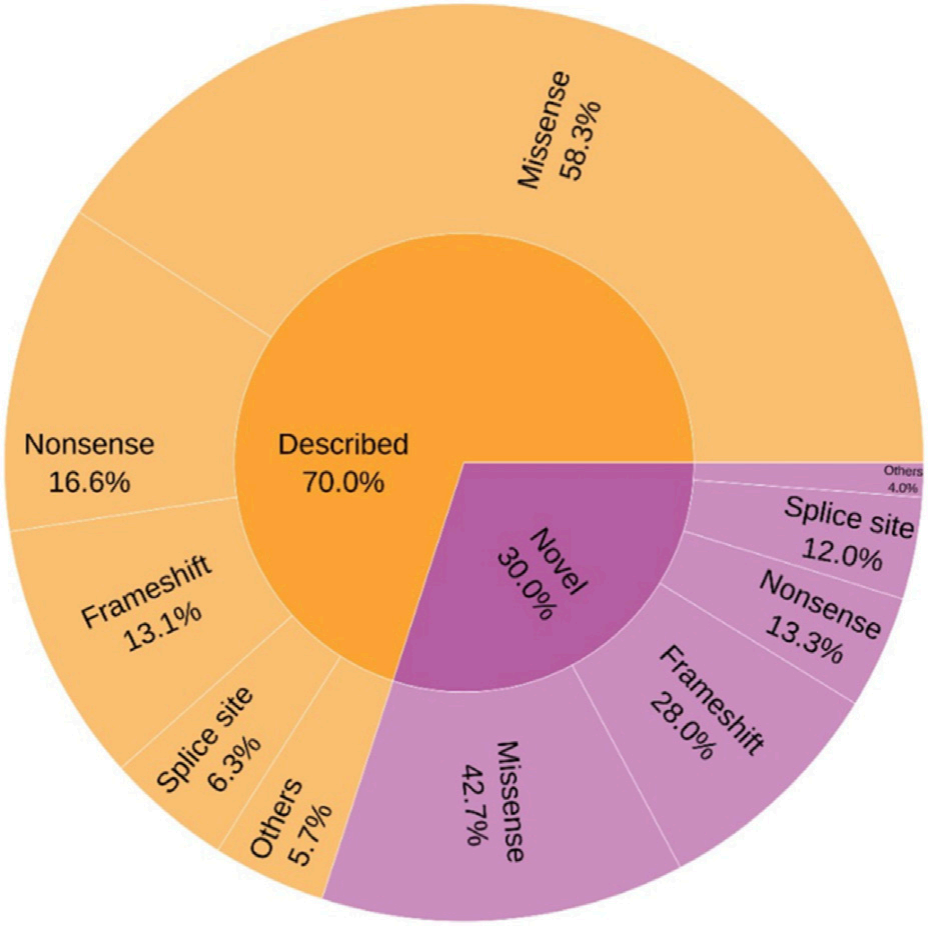
Multilevel donut chart showing the distribution of variants according to novelty and functional class. The inner ring represents variants classified as novel or previously described based on their presence in public databases and the scientific literature, whereas the outer ring indicates variants functional classes. Missense variants represent the largest proportion, followed by loss-of-function variants (nonsense, frameshift and splice-site alterations). The remaining fraction comprises other variant types, including mitochondrial, start-loss, synonymous, non-coding and in-frame variants.

Variants identified were distributed as novel or described, based on their presence in public databases and scientific literature, and further categorized according to their functional consequence (Figure 2). Missense variants represented the most frequent functional class in both groups, followed by loss-of-function variants, including nonsense, frameshift and splice-site alterations. A smaller fraction comprises in-frame insertions/deletions, mitochondrial changes, start loss, synonymous and non-coding variant.

Causative variants were most frequently associated with autosomal dominant inheritance (n = 91; 61%), followed by autosomal recessive (n = 40; 27%), X-linked (n = 10; 7%), and mitochondrial inheritance (n = 1; 0.7%). Additionally, seven cases (4.7%) involved conditions compatible with either autosomal dominant or autosomal recessive patterns. Among autosomal recessive cases, 57.5% (n = 23) were homozygous and 42.5% (n = 17) were compound heterozygous, with phase confirmation available for approximately half of these cases.

Familial segregation analysis by Sanger sequencing was performed in 28 of the 149 positive cases,18 autosomal dominant and 10 autosomal recessive cases, providing support evidence for variant pathogenicity. Among inconclusive cases, segregation analysis was conducted in 7 of the 63 cases; however, the available familial data did not provide sufficient evidence to support reclassification of the variant.

Systematic evaluation for ACMG-recommended secondary findings was limited to WES cases (Miller et al., 2023). Eight individuals (3.2%) harbored reportable pathogenic or likely pathogenic variants, all of whom had consented to receive such information. Clinically actionable findings included variants in cardiac-associated genes (*MYBPC3*, *TNNI3*), cancer predisposition genes (*BRCA2*, *APC*), genes associated with anesthesia-related risk (*RYR1*), and genes implicated in vascular anomalies (*ENG*, *ACVRL1*). Notably, identification of a pathogenic *BRCA2* splice-site variant prompted cascade testing, revealing maternal inheritance and enabling timely referral for specialized oncologic evaluation.

An important incidental finding emerged in a patient initially referred primarily due to primary ciliary dyskinesia. Following the detection of a known pathogenic *FGFR3* variant, a comprehensive re-evaluation of clinical features identified previously undocumented patient with short stature consistent with hypochondroplasia, underscoring the value of detailed phenotypic reassessment in genomic diagnostics.

## Discussion

The present study describes the feasibility and clinical impact of implementing NGS within a public reference service for rare diseases within the Brazilian SUS. Over 5 years, the incorporation of CES and WES enabled earlier and more accurate molecular diagnoses, supporting clinical management, guiding genetic counseling, and reducing the prolonged diagnostic odyssey commonly experienced by individuals with rare diseases. The overall diagnostic yield of 38.7% represents a substantial improvement over conventional diagnostic approaches and underscores the value of genomic testing as a public health tool. These findings parallel diagnostic yields reported in high-income countries (Clark et al., 2018; Splinter et al., 2018; Álvarez-Mora et al., 2022; Andersen et al., 2025), emphasizing the relevance of NGS-based diagnostics even in resource-constrained settings.

Diagnostic yields varied markedly across clinical groups, with conditions characterized by specific and well-defined clinical manifestations achieving higher rates of resolution than phenotypically heterogeneous disorders. Syndromic patient’s category yielded a diagnostic rate of 49.4%, while neurodevelopmental disorders group (NDD) showed a considerably lower yield (23.6%). This trend aligns with previous rates indicating that the diagnosis of NDD, including intellectual disability, global developmental delay and autism spectrum disorder; is particularly challenging due to substantial clinical and etiological heterogeneity. WES typically achieves diagnostic yields of approximately 30% in such cohorts, with higher yields observed when additional features are present (Srivastava et al., 2019; Carter et al., 2023; Thevenon et al., 2016). In our study, many individuals presenting with intellectual disability accompanied by syndromic features were classified within the congenital anomalies/syndromic group, which may partly account for the observed differences between categories.

Systematic reviews and meta-analyses report that WES diagnostic yields typically range from 25% to 40%, depending largely on patient selection and cohort characteristics (Seo et al., 2020; Slavotinek et al., 2023; Srivastava et al., 2022). In our service, CES demonstrated a higher diagnostic yield than WES (50% vs. 31%), primarily reflecting the stringent case selection applied during the early stages of NGS implementation, when testing focused on well-characterized and relatively homogeneous clinical groups, particularly RASopathies and other conditions with defined molecular etiologies. This clinical enrichment likely introduced a referral-related bias and should not be interpreted as evidence of intrinsic superiority of CES over WES. Rather, these findings highlight that CES can achieve high diagnostic yields in carefully selected clinical contexts. As the diversity and complexity of referred cases increased and the limitations of panel-based approaches became evident, WES was incorporated into routine diagnostics in 2023, enabling more comprehensive evaluation of heterogeneous phenotypes and supporting more effective clinical management and genetic counseling. In addition, the progressive reduction in NGS costs has favored broader adoption of WES in clinical practice, including the use of virtual gene panels derived from exome data, which allow targeted analysis while preserving the possibility of future reanalysis and broader genomic investigation (Molina-Ramírez et al., 2021).

A key strategy contributing to diagnostic success was the close integration between clinical and laboratory teams, fostering a multidisciplinary framework for genomic analysis. This model incorporated detailed phenotypic annotation using HPO terms, comprehensive clinical information provided at the time of test request, and review of previous genetic testing results, including chromosomal microarray (CMA) and targeted gene panels. These practices facilitated effective variant prioritization and minimized the risk of misinterpretation by filtering out findings unrelated to the patient’s phenotype. Although not quantitatively assessed in this study, our experience aligns with evidence demonstrating that genomic multidisciplinary team (MDT) models enhance diagnostic yield and improve patient care, with reported increases of 6%–25% in diverse clinical settings (Lazaridis et al., 2016; Rupp et al., 2019; Marinakis et al., 2021). MDT approaches have also been shown to support the resolution of VUS and to promote genomic mainstreaming by increasing genomic literacy among non-genetics clinicians (Vadlamudi et al., 2022).

Pre-test genetic counseling was essential to ensure that patients and families understood the purpose of testing (diagnosis, reproductive implications, clinical management), technical limitations (coverage gaps, false negatives), and the potential for identifying VUS or incidental and secondary findings (Elliott and Friedman, 2018). Evidence indicates that structured pre-test consultations improve patient understanding, support informed decision-making, and reduce post-test regret and stress (Gereis et al., 2022).

Accurate and standardized phenotypic description at the time of test request is crucial for effective genomic analysis. The HPO provides a hierarchical, structured vocabulary widely adopted in genomic medicine to support computational diagnostics and data interoperability (Robinson et al., 2008; Köhler et al., 2019). However, in this study, the use of HPO terminology varied substantially, reflecting broader challenges in clinical practice, particularly among non-geneticist physicians. Although retrospective curation was performed, 33 cases, mostly referred from external services, remained without standardized HPO annotation. These observations highlight the need for targeted training initiatives to improve genomic test ordering and to reinforce the importance of phenotype-driven variant prioritization for diagnostic accuracy. When available, clinicians should select the most specific HPO terms, as detailed annotations improve variant prioritization and downstream analyses. Although there is no consensus on the minimum number of HPO terms required, previous studies suggest that five well-selected terms represent a practical threshold for exome interpretation (Kernohan et al., 2018; Köhler et al., 2019).

The distribution of recurrent HPO terms across nosological groups also illustrates the challenges of summarizing phenotypic features in heterogeneous cohorts. The large size and hierarchical structure of the HPO, combined with substantial intra- and inter-group variability, limit the interpretability of frequency-based associations. Nonetheless, neurodevelopmental features, such as intellectual disability, developmental delay, and epilepsy, were overrepresented in the syndromic group, consistent with literature indicating that rare genetic disorders frequently affect developmental and neurological systems (Licata et al., 2023). This pattern likely reflects both referral characteristics and the clinical features that commonly prompt genetic evaluation in the Brazilian public healthcare system.

The identification of 75 novel variants expands the mutational spectrum of rare diseases in an underrepresented population and illustrates key challenges in variant interpretation. Large population databases indicate that each individual carries numerous rare coding variants, including an average of 27 ± 13 variants absent from other gnomAD individuals, particularly in populations with limited representation (Gudmundsson et al., 2022).

The Brazilian population is characterized by a highly admixed genetic structure resulting from historical contributions of European, African, and Indigenous ancestries. This complex demographic history likely contributes to the detection of a substantial number of previously undescribed variants and highlights the challenges of variant interpretation in populations that remain underrepresented in global genomic databases. The limited representation of such admixed populations further complicates variant interpretation and may increase the proportion of VUS. Thus, absence from population databases is insufficient to infer pathogenicity and contributes to the substantial burden of VUS (Naslavsky et al., 2021). In our cohort, 33 of the novel variants remained classified as VUS, contributing to 16% of cases being considered inconclusive. To mitigate misinterpretation, VUS reporting in our workflow includes specialist clinical review to assess the relevance of the implicated gene in the context of the patient’s phenotype. Nevertheless, definitive classification often requires functional assays, segregation analyses, and longitudinal follow-up. Although most VUS are eventually reclassified as benign or likely benign (Mighton et al., 2026), reclassification typically occurs slowly and rarely yields immediate clinical benefit. These findings underscore the need for expanded population-specific genomic databases, broader data sharing, and the integration of complementary interpretative strategies, such as mutational constraint metrics, functional genomics, expert-curated ClinGen guidelines, and periodic case reanalysis, to improve VUS resolution (Burke et al., 2022). Additionally, future incorporation of systematic structural and copy number variation analyses, which were not performed in the present study, may further improve diagnostic resolution in unresolved cases.

Secondary findings were identified in 3.2% of cases, highlighting the preventive potential and broader clinical utility of comprehensive genomic testing. These findings also underscore key ethical considerations related to result disclosure and the need for appropriate clinician training to ensure effective counseling and management. Incidental findings are an inherent aspect of comprehensive genomic analysis. In our cohort, one such finding led to the diagnosis of hypochondroplasia, initially unsuspected, in a patient referred for evaluation of primary ciliary dyskinesia, illustrating how careful phenotypic reassessment and close laboratory–clinical communication can significantly influence final diagnoses and subsequent care.

The successful implementation of NGS in our service required coordinated multidisciplinary efforts, sustained investment from institutional leadership (particularly through continuous investment in supplies and team qualification) and close engagement with patients and families to support genetic counseling. Despite ongoing challenges including cost constraints, interpretative complexity, and integration into routine clinical workflows this initiative demonstrates the transformative potential of genomics in public health. By enabling earlier and more accurate diagnoses, supporting personalized management, and generating population-relevant genomic data, the incorporation of NGS strengthens rare disease care and contributes to more equitable access to genomic medicine across Brazil.

## Conclusion

This report indicates that NGS can be successfully integrated into Brazil’s public healthcare system when supported by strategic resource allocation and specialized technical expertise. Despite operating under significant budgetary constraints, the establishment of a trained multidisciplinary team in genomics enabled diagnostic yields comparable to those achieved in high-income settings, contributing to shorter diagnostic delays and more informed clinical decision-making for individuals with rare diseases.

Beyond providing molecular diagnoses, the incorporation of genomic testing enhanced preventive care and genetic counseling, including the identification of actionable secondary findings and the refinement of clinical diagnoses through careful phenotypic integration. These outcomes illustrate how precision medicine can be effectively leveraged within public health systems to improve patient outcomes while promoting more efficient use of healthcare resources.

Furthermore, the concentration of diagnosed cases within the Southeast region of Brazil underscores persistent regional inequities in access to genomic services, an important barrier that must be addressed to achieve national equity in rare disease care. The experience presented here offers a scalable and context-appropriate framework for low- and middle-income countries, emphasizing the value of targeted workforce training, context-adapted testing strategies, and the integration of genomics into routine clinical pathways. Expanding such initiatives equitably across regions remains a critical challenge, but also a necessary step toward establishing genomic medicine as a sustainable and inclusive component of Brazilian public healthcare.

## Data Availability

The raw data supporting the conclusions of this article will be made available by the authors, without undue reservation.The datasets presented in this study can be found in online repositories. The names of the repository/repositories and accession number(s) can be found in the article/supplementary material.
